# Identification and Characterization of Alternative Splicing Variants and Positive Selection Genes Related to Distinct Growth Rates of Antlers Using Comparative Transcriptome Sequencing

**DOI:** 10.3390/ani12172203

**Published:** 2022-08-26

**Authors:** Pengfei Hu, Zhen Wang, Jiping Li, Dongxu Wang, Yusu Wang, Quanmin Zhao, Chunyi Li

**Affiliations:** 1 Institute of Antler Science and Product Technology, Changchun Sci-Tech University, Changchun 130600, China; 2 College of Traditional Chinese Medicine, Jilin Agricultural University, Changchun 130118, China

**Keywords:** antler growth rate, alternative splicing variants, positive selection genes, single molecule real time sequencing, comparative transcriptome

## Abstract

**Simple Summary:**

The size of antlers varies among species; antlers of the wapiti (*Cervus canadensis xanthopygus*) grow much faster than those of its close relative the sika deer (*Cervus nippon hortulorum*) in the same growing period. This contrast provides a potential model for comparative studies for the identification of potent growth factors and unique regulatory systems. In the present study, the reference transcriptomes of the antler reserve mesenchyme (RM) tissue of wapiti and sika deer were constructed using single molecule real time sequencing data. The expression profiling, positive selection, and alternative splicing of the antler transcripts were compared, and interactive relationships and expression patterns of hub genes were identified and analysed. We identified that *RNA Binding Motif Protein X-Linked* (*RBMX*) gene was under strongly positive selection. One gene found to interact with *RBMX* was *methyltransferase-like 3* (*METTL3*), an oncogene that could promote translation of cancer cell proteins. There was a contrasting relationship in expression level between *RBMX* and *METTL3* genes in the RM tissue. We believe our study can provide a better understanding of rapid antler growth at the molecular level in particular and endochondral ossification in general.

**Abstract:**

The molecular mechanism underlying rapid antler growth has not been elucidated. The contrast of the wapiti and sika deer antler provides a potential model for comparative studies for the identification of potent growth factors and unique regulatory systems. In the present study, reference transcriptomes of antler RM tissue of wapiti and sika deer were constructed using single molecule real time sequencing data. The expression profiling, positive selection, and alternative splicing of the antler transcripts were compared. The results showed that: a total of 44,485 reference full-length transcripts of antlers were obtained; 254 highly expressed transcripts (HETs) and 1936 differentially expressed genes (DEGs) were enriched and correlated principally with translation, endochondral ossification and ribosome; 228 genes were found to be under strong positive selection and would thus be important for the evolution of wapiti and sika deer; among the alternative splicing variants, 381 genes were annotated; and 4 genes with node degree values greater than 50 were identified through interaction network analysis. We identified a negative and a positive regulator for rapid antler growth, namely *RNA Binding Motif Protein X-Linked (RBMX)* and *methyltransferase-like 3 (METTL3)*, respectively. Overall, we took advantage of this significant difference in growth rate and performed the comparative analyses of the antlers to identify key specific factors that might be candidates for the positive or negative regulation of phenomenal antler growth rate.

## 1. Introduction

Deer antlers are unique mammalian bony organs that are periodically lost and regenerated and have an extraordinary growth rate [1]. As the most important weapon for fighting during rutting season, antlers are the products of sexual selection in the evolution of deer [2]. Antlers (especially from sika deer and wapiti) are also widely used as health products in traditional Chinese medicine in East Asia [3]. Their rapid growth in particular, has attracted much attention to antlers [1,4,5,6]. Research has shown that it is the rapid proliferation and differentiation of antler tip cells that drives antlers to grow so rapidly with differentiation towards chondrogenesis, maintaining pace during the further differentiation and growth of the antler [1,7,8,9]. Histologically, the antler growth centre is located in the antler tip and consists of four zones at different differentiation stages disto-proximally: reserve mesenchyme (RM), precartilage, transition and cartilage zones. RM cell proliferation and chondrocyte hypertrophy are the main drivers for rapid antler elongation [10]. Therefore, antlers could be used as an ideal model for studying the extraordinarily fast growth of endochondral bone formation.

Factors involved in endochondral ossification, especially those that participate in the proliferation of mesenchymal cells and the differentiation of chondroblasts to chondrocytes, play a very important role in the regulation of antler growth rate [11]. However, only a few have been identified, including *parathyroid hormone* (*PTH*)/*parathyroid hormone-related protein* (*PTHrP*) *receptors*, *insulin-like growth factor-I* and *testosterone receptors* [12,13,14,15,16]. It is known that many transcriptional factors are involved in endochondral ossification, including *runt-related transcription factor 2*, *Osterix*, *activating transcription factor 4*, *suppressor of cytokine signalling 1*, *special AT- rich sequence binding protein 2*, etc. The differentiation of chondroblasts and osteoblasts can also be induced by multiple cytokines and enzymes, such as *bone morphogenetic proteins*, *transforming growth factor-β family*, *fibroblast growth factors* and *methyltransferases*, etc. [17]. However, these are all generic growth promoting factors, and specific factors that regulate antler growth (positively or negatively) have, thus far, not been identified.

In order to reveal the underlying mechanism of rapid antler growth, a number of molecular biology approaches have been applied, such as cDNA chip microarray [18,19,20], cDNA library [21] and Illumina sequencing [22,23]. The results from these studies confirmed that antler growth is regulated by numerous factors reportedly involved in endochondral ossification. However, among the influencing factors, which ones play the most critical role remains uncertain.

Wapiti and sika deer are closely-related species and very similar at the genome level, with the separation of the two species estimated at only 2.7–4.7 million years [24]. They also hybridize with each other to produce fertile offspring. The wapiti is substantially larger than sika and the antlers of wapiti grow much faster than those of sika during the similar growing period [1]. Therefore, in the present study we took advantage of this significant difference in growth rate and performed comparative analyses of the antlers to identify key specific factors that might be candidates for the positive or negative regulation of phenomenal antler growth rate.

We compared the differences of antler tips (the antler growth centre) of wapiti and sika deer at transcriptional level during the rapid antler growth phase using single molecule real time (SMRT) sequencing. Differential expression, positive selection and alternative splicing transcripts were detected, and genes related to endochondral ossification and metabolism process were identified and characterized through interaction networks and expression pattern analyses. We believe our study will enable us to have a better understanding at the molecular level of rapid antler growth in particular and endochondral ossification in general.

## 2. Materials and Methods

### 2.1. Ethics Statement

All procedures related with animals were conducted to accord with the guidelines of care and use of experimental animals established by the Ministry of Agriculture of China, and all protocols were approved by the Institutional Animal Care and Use Committee of Institute of Antler Science and Product Technology, Changchun Sci-Tech University, Changchun, China (Ethics No.: CKARI202004).

### 2.2. Data and Sample Collection

The SMRT sequencing and Illumina sequencing data of growing antler tips from wapiti and sika deer were downloaded from NCBI (https://www.ncbi.nlm.nih.gov/sra/PRJNA531332 (accessed on 20 November 2021)), which were deposited previously by us. Deer samples were collected from deer of the same age (5 years old), raised under the same conditions and fed the same diet. The antlers used for sequencing were all in the rapid growth period (70 days of growth). It is recorded that the average tissue deposition of wapiti antler was 137 g/d and of sika deer it was 69 g/d (calculated by weighing from the time when antlers start to grow to 70 days).

RM tissue samples in the antler growth centre of the main beam were collected from the following deer with different antler growth status, respectively, on 70 days of growth (the most rapid growth period for these species): (1) sika deer with fast antler growth rate status (*n* = 3, 93 ± 5 g/d); (2) sika deer with slow antler growth rate status (*n* = 3; 47 ± 3 g/d); and (3) wapiti with fast antler growth rate status (*n* = 3; 137 ± 8 g/d), respectively (Table 1). The antler tips were removed, and RM tissues were sampled and frozen at −80 °C until use.

Six other parts of growing antlers and 12 types of control tissue were collected from three sika deer at slaughter: second branch RM, main branch cartilage, second branch cartilage, mid part antler, first branch antler, proximal region antler, cerebrum, cerebellum, heart, liver, kidney, rumen, lungs, spleen, small intestine, compact bone, cartilage and periosteum. The samples were frozen at −80 ℃ until use.

General anaesthesia was used during tissue or blood sampling (Lumianning, 070011777, Jilin Huamu Animal Health Products Co., Ltd., Changchun, China) via intramuscularly injection at 1 mL/100 kg body weight. Deer were then either released or slaughtered after tissue or blood sampling.

**Figure 1:**
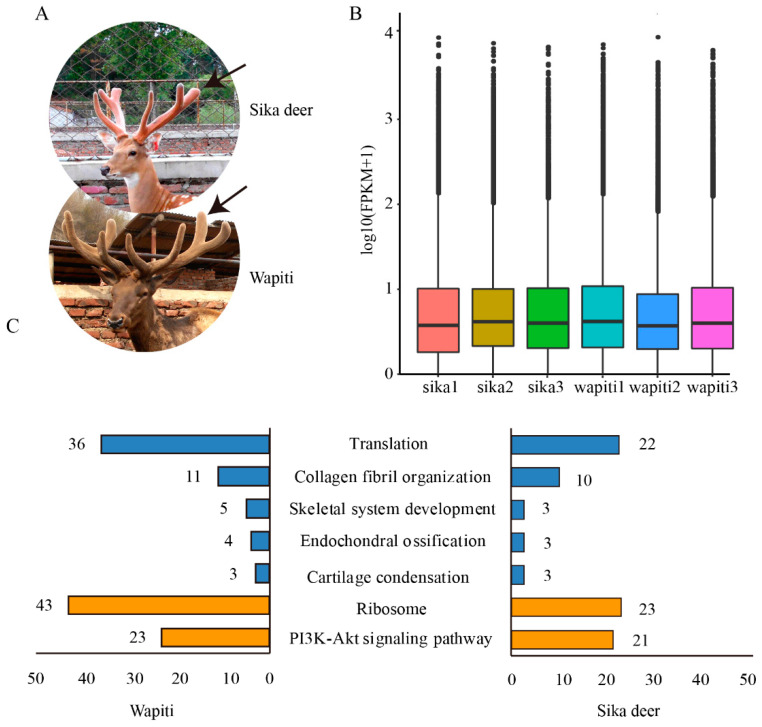
Gene expression and enrichment analyses of RM tissue in the antler growth centre of wapiti and sika deer: (**A**) Sika deer and wapiti antlers with the same growth stage. Tissues were sampled from the growing tips of antler (arrows). (**B**) Box drawing of all transcripts in the RM tissue of after FPKM data standardization. (**C**) Functional enrichment analysis of the highly expressed genes between wapiti and sika deer. Enriched GO terms (represented using blue columns) and signalling pathways (represented using orange columns) were highly correlated with translation, endochondral ossification and ribosome.

### 2.3. Reference Transcriptome Construction of Antlers

The raw SMRT sequencing data were analysed using SMRT Link 4.0 software to obtain the full-length transcripts of wapiti and sika deer antlers, respectively. Error corrections of the full-length transcripts were conducted using Illumina data PROVE READ software, redundant transcripts were removed using a cd-hit-est software. The finally obtained transcripts were applied for gene annotation and CDS prediction.

The length and number of full-length transcripts of wapiti and sika deer were compared. Based on k-mer similarity, full-length transcripts of wapiti and sika deer were clustered to different transcript families using Coding GENome reconstruction Tool (Cogent 3.1), respectively, and each transcript family was further reconstructed into one or several unique transcript model(s) using a De Bruijn graph method. The reconstructed transcripts were defined as the reference transcriptome of antlers.

### 2.4. Analysis of Gene Expression in Wapiti and Sika Deer Antlers

Clean reads of Illumina data of each sample were mapped to the reference transcriptome of antlers using RSEM software, the read counts on each transcript were obtained by comparing each sample to the reference transcriptome and converted to Fragments Per Kilobase of exon model per million mapped fragments (FPKM represents the relative expression levels of genes). A box map of the gene expression level of each sample was drawn based on FPKM value.

Highly expressed transcripts (HETs) and differentially expressed genes (DEGs) between wapiti and sika deer antlers were identified using DESeq software analysis. The threshold for screening DEGs was “padj < 0.001”. The Omicshare Tools (https://www.omicshare.com/tools/Home/Soft/heatmap (accessed on 15 December 2021)) were used to conduct cluster analysis of the DEGs.

### 2.5. Gene Function Enrichment Analysis

The DAVID 6.8 (https://david.ncifcrf.gov/ (accessed on 15 December 2021)) tool was used for the GO and KEGG enrichment analysis of the obtained gene sets, *p* value < 0.05 was considered as significantly enriched. The enriched GO terms were mapped to networks according to the order of hierarchy and shown using a bubble network diagram through Cytoscape 3.6.0 software. The enriched KEGG pathways were visualized with the seniorbubble diagram using Omicshare Tools (https://www.omicshare.com/tools/Home/Soft/seniorbubble (accessed on 15 December 2021).

### 2.6. Orthologous Gene Analysis

Orthologous genes were identified from the full-length CDS sequences of wapiti and sika deer antlers using OrthoMCL software, synonymous substitution and non-synonymous substitution of these genes were calculated using paml-codeml software. The ratio of non-synonymous substitution rate (Ka) to synonymous substitution rate (Ks), namely Ka/Ks, was also calculated. Selective pressure on the coding genes was judged based on the ratio of Ka/Ks. If Ka/Ks >>1, the gene was defined as divergent gene and we would speculate that this is a positive selection, which is very important for species evolution. The divergent genes were used for gene function enrichment analysis using DAVID 6.8 tool.

### 2.7. Alternative Splicing Analysis

Error-corrected non-redundant transcripts (transcripts before reconstruction) were mapped to the reference transcriptome of antlers using GMAP software. Splicing junctions for transcripts mapped to the same full-length transcripts were examined, and transcripts with the same splicing junctions were collapsed. Collapsed transcripts with different splicing junctions were identified as the transcriptional isoforms of the reference full-length transcripts. The alternative splicing events were detected using SUPPA software (https://github.com/comprna/SUPPA (20 December 2021)) on default settings. The exon–intron structures of the transcriptional isoforms were identified using GSDS 2.0 software (http://gsds.cbi.pku.edu.cn/ (accessed on 22 December 2021).

### 2.8. Gene Interaction Network Analysis

The interactions of all the DEGs between wapiti and sika deer antlers were analysed using the STRING database (https://string-db.org (accessed on 22 December 2021) version 11.0), cattle genome was taken as a reference and the confidence value set at 0.9. The file of gene interactions was imported into Cytoscape 3.6.0 software for creation of the visualization style of gene interaction network. The Cytoscape plugin, ClusterOne, was used for the sub-network module analysis, and genes in the significant enriched modules were taken for further functional enrichment analysis. The Cytoscape plugin, CytoHubba was used to identify the hub genes within the gene interaction network; genes with a degree value greater than 50 and closely related to endochondral ossification and metabolism process were considered hub genes.

### 2.9. Gene Expression Profiling in Different Tissue Types from Antlers with Different Growth Rate

Firstly, ten DEGs were randomly selected for verification using quantitative real-time PCR (qPCR). Total RNAs were extracted from the RM tissue samples of the antler growth centre of wapiti and sika deer, respectively. Primers were designed using NCBI primer-BLAST software, *Beta-actin* (*ACTB*) was used as an internal reference, gene expression was analysed on a Roche 480 Real Time PCR (qPCR) System using LightCycler 480 SYBR Green I Master (Roche Pharmaceutical Ltd., Basel, Switzerland).

Subsequently, hub genes identified from the gene interaction network analysis were selected for gene expression analysis in different tissue types from antlers of the different growth rate classes of sika deer. Total RNAs were extracted from the different tissue types of the different growth rate antlers. The expression of the hub genes was measured using qPCR as described above.

The qPCR results were analysed using the 2^−^^ΔΔ^^CT^ method, the results presented as means ± SE and then visualized using GraphPad Prism 8 software.

## 3. Results

### 3.1. Reference Transcriptome and Gene Expression Profiling of Antlers

Although both wapiti and sika deer belong to the same genus Cervus, the growth rates of their antlers are significantly different (Figure 1A). To construct the reference transcriptome of antlers for each deer species, full-length transcripts from both wapiti and sika deer antlers were assembled and compared. A total of 317,149 consensus reads were obtained from the SMRT sequencing dataset of wapiti after correction and de-redundancy, and 24,970 full-length transcripts obtained with average length of 1658 bp. The full-length transcripts of wapiti antlers were comparable to the full-length transcripts of sika deer (Table S1), as also shown in previous work [24], and the expression levels of these full-length transcripts were very similar (Figure 1B). After de-redundancy and transcript reconstruction of both full-length transcripts of wapiti and sika deer, the reference transcriptome was constructed with a total of 44,485 full-length transcripts of antlers (Table S2).

The gene expression profiles of the wapiti and sika deer antlers were analysed based on the reference transcriptome of antlers. A total of 254 HETs (read count > 10,000) in wapiti and 253 HETs (read count > 10,000) in sika deer were obtained (Table S3). The functional enrichment analysis of the HETs in wapiti and sika deer showed that many enriched GO terms and signalling pathways were highly correlated with rapid growth and metabolism, such as biological process of translation, collagen fibril organization, skeletal system development, endochondral ossification and the signalling pathways of ribosome and PI3K-Akt (Figure 1C).

**Figure 2:**
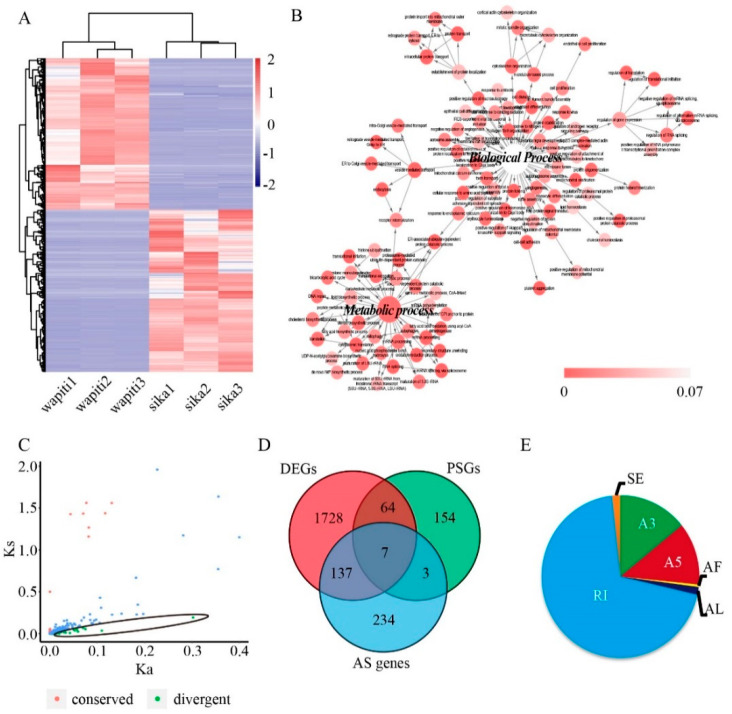
Differential expression, positive selection and alternative splicing events in RM tissue in the antler growth centre of wapiti and sika deer: (**A**) Heatmap of 3899 differential expression transcripts. The map was drawn through comparison of read count on each transcript of wapiti and sika deer. Of these transcripts, 1882 were up−regulated in the wapiti antler and 2017 up−regulated in sika deer antler. (**B**) The functional enrichment analysis of differentially expressed genes (DEGs). Note that most of the significantly enriched biological processes were found to all be related with the metabolism. (**C**) Distribution of Ka and Ks of 1531 orthologous transcripts. Of these transcripts, 315 were found to be divergent transcripts (Ka/Ks > 1). (**D**) Venn diagram of DEGs, positive selection genes (PSGs) and alternative splicing genes (AS genes). (**E**) The type and distribution of the alternative splicing events: 448 intron retention (RI) events accounting for 69.67% of the total alternative splicing events, 91 alternative 3’ splice site (A3), 80 alternative 5’ splice site (A5), 11 exon skipping (SE), 3 alternative first exon (AF) and 10 alternative last exon (AL), respectively.

In total, 3899 DETs were obtained after the comparison of read counts on transcripts of each species (Figure 2A, Table S4). Among these transcripts, 1882 were up-regulated in wapiti antlers and 2017 up-regulated in sika deer antlers (Table S4). After gene annotation, a total of 1936 DEGs were identified (Table S5). The results of functional enrichment analysis of these DEGs showed that most of the significant enriched biological processes were all correlated with metabolism (Figure 2B) and many biological processes were related to endochondral ossification (Table S6). The significant enriched signalling pathways mainly included metabolic pathways and ribosome (Figure S1). The enrichment of HETs and DEGs in the same biological process and signalling pathways correlated with the metabolism process and endochondral ossification, suggesting that these biological processes and signalling pathways play an important role in the regulation of rapid antler growth. Ten of these genes were randomly selected for qPCR verification (Table S7), and the results showed that gene expression patterns were consistent with those of SMRT sequencing (Figure S2), providing strong evidence that our SMRT sequencing results are reliable.

### 3.2. Characterization of Orthologous Genes of Wapiti and Sika Deer Antlers

To characterize orthologous genes of wapiti and sika deer antlers, gene annotation and CDS prediction from the full-length transcripts were carried out; a total of 21,871 CDS were identified in wapiti and 51,740 CDS in sika deer, respectively. Orthologous transcripts were searched against these CDS sequences, and 1531 orthologous transcripts matched (Table S8). Among these matched transcripts, 315 were divergent transcripts (Ka/Ks > 1; Figure 2C). After annotation, 228 divergent genes were identified and there were numerous non-synonymous substitutions in these divergent genes, indicating that they were under strong positive selection, which could be evidence of evolutionary differences between wapiti and sika deer. The results of the functional enrichment analysis of the 228 divergent genes showed that significantly enriched biological processes were collagen fibril organization and cell growth (Table S9), and the significant enriched signalling pathways were ribosome and metabolic pathways (Table S9). Of the 228 divergent genes between wapiti and sika deer, 71 were DEGs in the RM zone of the antler growth centres (Figure 2D).

### 3.3. Alternative Splicing Events in the Wapiti and Sika Deer Antlers

To detect alternative splicing events in the antlers, a total of 3615 isoforms were identified from the reference transcriptome, which contains 44,485 full-length transcripts (Table S10), indicating that isoforms were abundant in the transcripts. Alternative splicing events were found at 643 sites from the 3615 isoforms using SUPPA software (Table S11). These results were further confirmed by using our Illumina data, indicating that these alternative splicing events were accurate. The type and distribution of these alternative splicing events were analysed. There were 448 intron retention (RI) events found to account for 70% of the total alternative splicing events; there were also 91 alternative 3’ splice sites (A3), 80 alternative 5’ splice sites (A5), 11 exon skipping (SE), 3 alternative first exon (AF) and 10 alternative last exon (AL), respectively, while there were no mutually exclusive exons encountered (MX) (Figure 2E).

A total of 381 genes were annotated from the alternative splicing isoforms; 144 (38%) were found to be differentially expressed in the RM zone of wapiti and sika deer antlers (Figure 2D). Of these 144 genes, seven (*RBMX, METTL3, TMEM219, LOXL2, LOC100128274, SENP2* and *NKTR*) were found to be under positive selection. After a detailed analysis of the isoforms of these seven genes, we found that alternative splicing resulted in the loss/gain of the gene function domain or the premature termination of transcription, which would eventually affect gene expression and function.

### 3.4. Characterization of Hub Genes Related with Regulation of Antler Growth Rate

**Figure 3:**
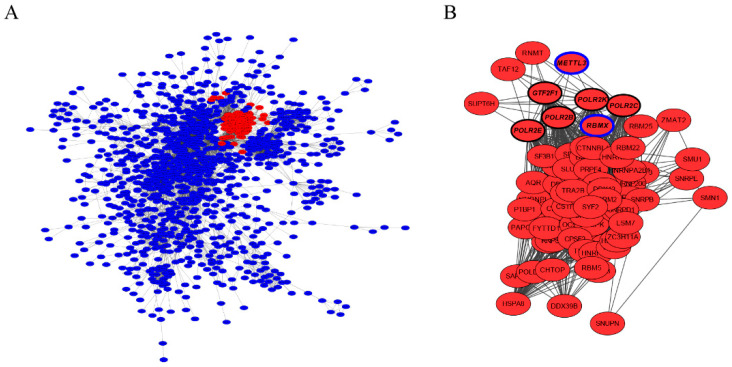
Gene interaction and sub-module analyses of DEGs in the RM tissue in the antler growth centre of wapiti and sika deer: (**A**) Interaction of DEGs in the antlers of wapiti and sika deer, the red sub module represents the most significant sub module (*p* value < 0.01). (**B**) The most significant sub module contains *RBMX*, which has a potential interaction with *METTL3* through the *transcription initiation factor* (*GTF2F1*) and *RNA polymerase II* (*POLR2E, POLR2K, POLR2C* and *POLR2B*).

To define hub genes involved in the regulation of antler growth rate, the interaction network of all the identified DEGs were analysed (Figure 3A), the node degree of each gene was calculated and presented in Table S12. In total, 71 genes were found to be differentially expressed and positively selected and have been sorted according to their node degree value. Of these genes, four with a node degree value larger than 50 were selected as hub genes, namely *UBB, CPSF2, SRSF3* and *RBMX*. Among these hub genes, *RBMX* was under strong positive selection (Table S8); the sub-network module analyses of all the DEGs showed that the *RBMX* module was the most significant module (Table S13). The functional enrichment analysis of genes in the *RBMX* module showed that these genes were mainly enriched in the metabolism process (Figure S3). *RBMX* could interact with *METTL3* through transcription initiation factor (*GTF2F1*) and RNA polymerase II (*POLR2E, POLR2K, POLR2C* and *POLR2B*) (Figure 3B).

**Figure 4:**
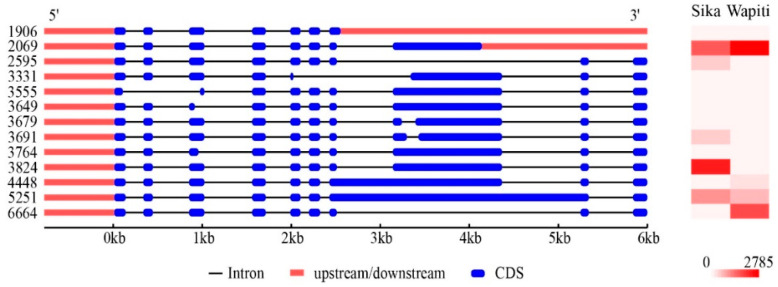
The exon–intron structures and expressions of 13 transcripts of *RBMX* gene. The exon-intron structures of *RBMX* and expression of the 13 transcripts of *RBMX* were compared between wapiti and sika deer antlers and transcript 6664 was found to be expressed specifically in wapiti antler, while transcript 3824 was expressed specifically in sika deer antler.

**Figure 5:**
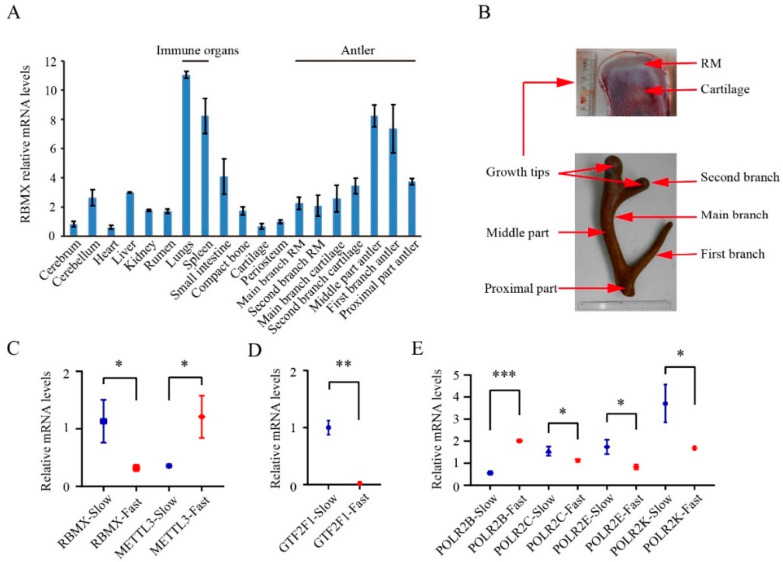
Characterization of *RBMX* and *METTL3* in growing antlers: (**A**) Expression levels of *RBMX* in seven segments of growing antlers and 12 types of control deer tissue: main branch RM zone, second branch RM zone, main branch cartilage zone, second branch cartilage zone, middle part antler, first branch antler, proximal part antler, cerebrum, cerebellum, heart, liver, kidney, rumen, lungs, spleen, small intestine, compact bone, cartilage and periosteum. (**B**) Sampling diagram of seven segments of growing antlers. (**C**) Expression levels of *RBMX* and *METTL3* in the antlers with fast and slow growth rate. (**D**) Expression levels of *GTF2F1* in the antlers with fast and slow growth rate. (**E**) Expression levels of *POLR2E, POLR2K, POLR2C* and *POLR2B* genes in the antlers with fast and slow growth rate. * *p* < 0.05; ** *p* < 0.01; *** *p* < 0.001; blue colour, antler with slow growth rate; red colour, antler with fast growth rate.

The *RBMX* gene contained two alternative splicing sites, namely A5 and RI (Table S11). The expression profiling of 13 transcripts of *RBMX* was further analysed. In total, seven expressed transcripts of *RBMX* were identified from the antlers. Among them, transcript 6664 was specifically expressed in the wapiti antler, whereas transcript 3824 was specifically expressed in the sika deer antler (Figure 4). The RBMX gene was highly expressed in the growing antlers and immune organs (Figure 5A,B).

The analysis of the expression profiling of *RBMX, METTL3, GTF2F1, POLR2E, POLR2K, POLR2C* and *POLR2B* genes in the RM tissues sampled from the fast and slow growing antlers of sika deer further demonstrated that *RBMX* was the gene that was more highly expressed in the slow-growing sika antlers; in contrast, *METTL3* was more highly expressed in the fast-growing sika deer antlers. The rest of the genes (*GTF2F1, POLR2E, POLR2K* and *POLR2C*), except *POLR2B*, had similar expression patterns to *RBMX* (Figure 5C–E) in the growing antlers. Overall, we identified a positive and a negative regulator for antler growth rate, i.e., *METTL3* and *RBMX*, respectively. *RBMX* was under strongly positive selection. *METTL3* could interact with *RBMX*. There was a contrasting relationship in expression level between *RBMX* and *METTL3* genes in the RM tissue. We believe our study would provide a better understanding of rapid antler growth at the molecular level in particular and endochondral ossification in general.

## 4. Discussion

Differences in gene expression profiles and alternative splicing in antlers of the two deer species and in antlers at differential growth rates in sika deer have been detected in the present study. Although both Illumina and SMRT sequencing of antler tip tissue have been reported in previous studies [23,25,26,27] for different growth stages of antlers, this study has greatly expanded knowledge in terms of sika (especially in terms of full-length transcripts and alternatively-spliced isoforms) and extended to a new deer species, wapiti. Both the comparison with wapiti and the comparison of sika deer antlers at differential growth rates have added a new dimension to the field. Moreover, many differentially expressed alternative splicing isoforms and divergent genes were first identified in the antlers through comparative analyses. These isoforms and divergent genes may provide clues to molecular mechanisms underlying differences in growth rate of antlers within and between species. At the same time, the reconstructed transcripts of wapiti and sika deer could be used as the reference transcriptome for subsequent antler biology studies.

Antler is a unique organ with soft hairy skin covering an osseocartilaginous tissue. Antlers are rich in blood vessels and nerves, with an unprecedented growth rate for a mammalian tissue. Differences between wapiti and sika deer antlers in terms of weight and branch number and their similarity in histological structure are well-known [4].

In our previous studies, many differentially expressed genes related to organ development processes were found in the faster growing antlers compared with slower growing antlers [11]. This study was designed to compare full-length transcripts of antler tissues of wapiti and sika deer, mainly in the aspects of transcript expression profiling and alternative splicing. There is evidence that alternative splicing events play an important role in organ development and the functional regulation of organism growth [28,29,30], but the information of isoforms of wapiti and sika deer antlers could help us to identify differentially expressed alternative splicing transcripts, which may be those that regulate the significant differences in the morphology and growth rate of antlers between wapiti and sika deer. Particularly, the DEGs and divergent genes were all significantly enriched in the metabolic pathways, the highly expressed genes were all related to endochondral ossification and the four hub genes were found to be involved in antler growth; all these results provide insight into the mechanism underlying the molecular regulation of antler growth.

A number of the hub genes identified were found to be associated with metabolism process and endochondral ossification. It was reported that the loss of *METTL3* gene function leads to impaired bone formation, reduced osteogenic differentiation and the downregulation of the *PTH* regulatory gene expression [31]. However, while this did not affect *PTH1R* gene expression, there was reduced *PTH1R* protein synthesis efficiency at the translational level and evidence of further interference with *PTH*-induced osteogenesis in vivo. In view of the important role of *PTH* in the regulation of antler growth [14,32,33], we speculated that *METTL3* may be a positive regulator of antler growth rate. However, *METTL3* expression was not detected in the chondrocytes of growth plate, so it may not directly influence chondrocyte differentiation [31]. In this study, we found that *METTL3* was highly expressed in the RM tissue of the fast-growing antlers, and the hub gene, *RBMX*, which could potentially interact with *METTL3*, exhibited the reverse expression pattern to *METTL3*.

The *RBMX* gene was found to be under strong positive selection between wapiti and sika deer in the present study, indicating that it is undergoing rapid evolution, which would be of great significance to the evolution of wapiti and sika deer. *RBMX* was initially called *hnRNP G* (*heterogeneous nuclear ribonucleoprotein G*), coded by an X-chromosome gene. It is reported that *RBMX* is amongst the earliest recruits to sex chromosomes over 130 million years ago [34], indicating that *RBMX* is an X-derived autosomal retrogene, which usually evolves with distinct functions [35,36,37]. *RBMX* proteins are implicated in splicing control particularly during the development of the nervous system [38]. Coincidentally, antlers have been reported to originate from neural crest stem cells [39]. Functional studies have shown that the *RBMX* gene is essential for embryo development, and the loss of the gene results in head and eye dysplasia, diminished body size, missing jaws, etc. [40]. In mice, the *RBMX* protein is highly expressed in peripheral nerves, and its expression in neurons from the central nervous system can promote axon and dendrite growth [41]. In rats, *RBMX* protein expression was increased after spinal and retinal injury [42]. For example, the *RBMX* gene, as a key regulator, is closely linked to important cancer drivers [43] with evidence that it may act as a tumour suppressor [44] and may help to protect the integrity of the genome from damage [45,46]. In the present study, we found that the *RBMX* gene interacts with many genes that were differentially expressed in between wapiti and sika deer antlers and had multiple alternative splicing transcripts. Importantly, some specific transcripts of *RBMX* were found to be differentially expressed in antlers of sika deer and wapiti (e.g., transcripts 6664 and 3824), which may have different roles in regulating antler growth rate and are thus worthy of further study. Because *RBMX* was downregulated in the faster growing antlers, we believe that *RBMX* gene is a key factor in the suppression of the antler growth rate.

## 5. Conclusions

In summary, we constructed a reference transcriptome for deer antlers, compared the expression profiling, assessed evidence for positive selection and alternative splicing transcripts of antlers in wapiti and sika deer. Our results revealed the interaction networks and expression patterns of hub genes. We found that *RBMX*, along with *METTL3*, are strong candidates as key factors for regulating antler growth rate.

## Figures and Tables

**Table 1:** Samples collected for qPCR verification.

	Number	Samples	Characteristic	Estimated Growth Rate of Antler
Live deer				
Sika deer	3	Tip of main beam (MB) of antler (Figure 1A)	Growth rate	93 ± 5 g/d
Sika deer	3		Growth rate	47 ± 3 g/d
Wapiti	3		Growth rate	137 ± 8 g/d
Slaughtered deer				
Sika deer	3	Antler: MB growth tip cartilage; second branch (SB) growth tip RM and cartilage; middle part of antler; first branch antler; proximal part of the antler		
		Other tissues: cerebrum, cerebellum, heart, liver, kidney, rumen, lungs, spleen, small intestine, compact bone, cartilage and periosteum		

## Data Availability

The raw data are available from the SRA (http://www.ncbi.nlm.nih.gov/sra/, accessed on 12 October 2021) data repository (accession number: PRJNA531332).

## Supplementary Materials

The following supporting information can be downloaded at: https://www.mdpi.com/article/10.3390/ani12172203/s1, Figure S1: The significant enriched signal pathways of differential expression genes between wapiti and sika deer velvet antler, Figure S2: Gene expression patterns between quantitative real-time PCR and SMRT sequencing, “**” represented 0.001 < *p* < 0.01, “*” represented 0.01 < *p* < 0.05, Figure S3: Functional enrichment analysis of genes in the most significant RBMX module, Table S1: Statistics of consensus reads, full length transcripts and Illumina reads in the developing velvet antler of wapiti and sika deer, Table S2: Cluster of all transcripts in the developing velvet antler of wapiti and sika deer, Table S3: High expression transcripts in the developing velvet antler of wapiti and sika deer, Table S4: Differential expression transcripts in the developing velvet antler of wapiti and sika deer, Table S5: Differential expression genes in the developing velvet antler of wapiti and sika deer, Table S6: GO enrichment of differential expression genes in the developing velvet antler of wapiti and sika deer, Table S7: Primer sequences for real-time PCR, Table S8: Orthologous genes of wapiti and sika deer velvet antler, Table S9: Enrichment analysis of divergent genes of wapiti and sika deer, Table S10: Isoforms in the developing velvet antler of wapiti and sika deer, Table S11: Alternative slicing events in the developing velvet antler of wapiti and sika deer, Table S12: Node degree of gene interaction network in the developing velvet antler of wapiti and sika deer, Table S13: Sub-network module of all the differential expression genes in the developing velvet antler of wapiti and sika deer.
