# Supplementary figures and images for: Identification and Characterization of Alternative Splicing Variants and Positive Selection Genes Related to Distinct Growth Rates of Antlers Using Comparative Transcriptome Sequencing
Source: Animals (Basel). 2022 Aug 26;12(17):2203. doi: 10.3390/ani12172203 (PMC9454627; doi:10.3390/ani12172203)

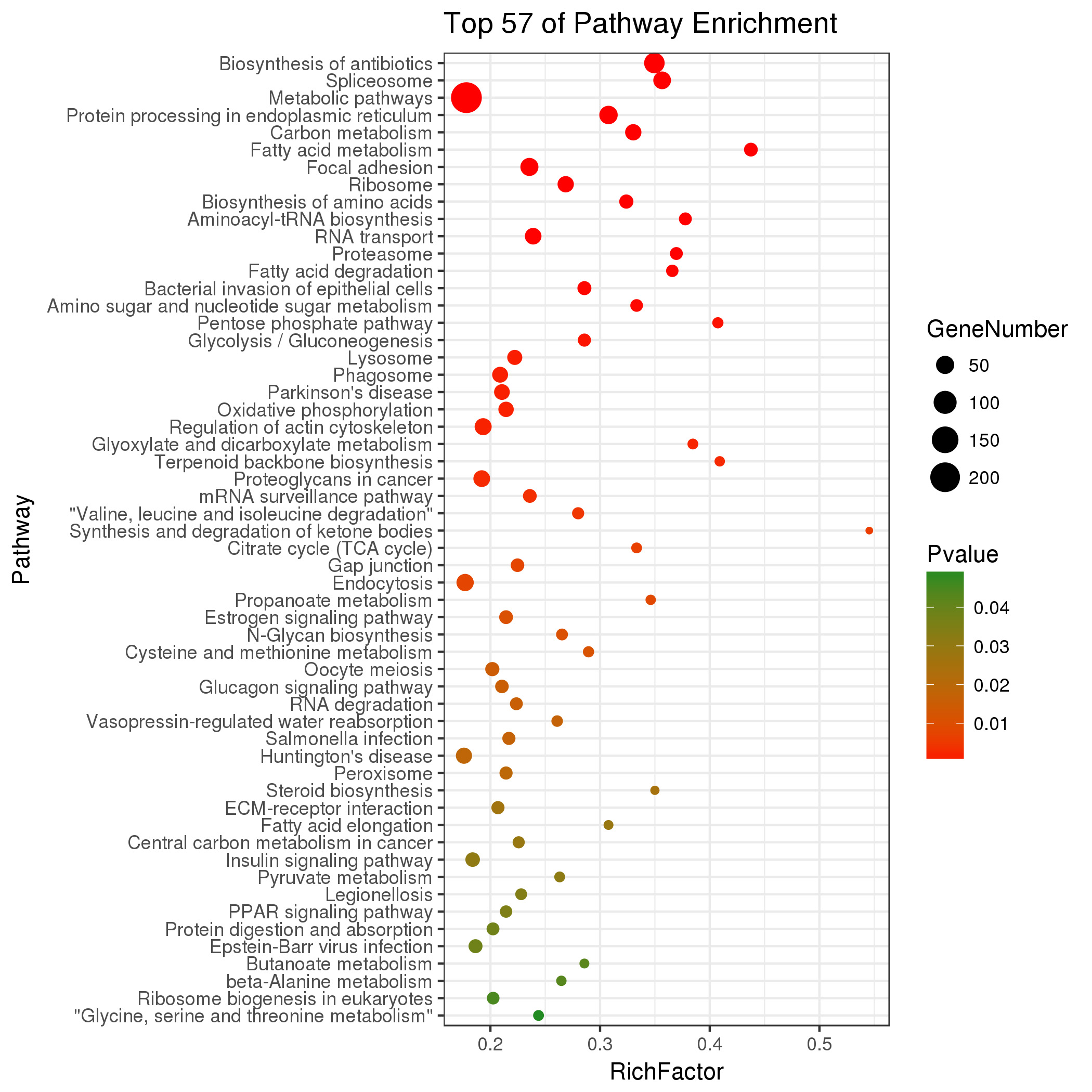

Supplement: Supplementary file 1 [file animals-12-02203-s001.zip › animals-1855021-supplementary/Figure S1.jpg]

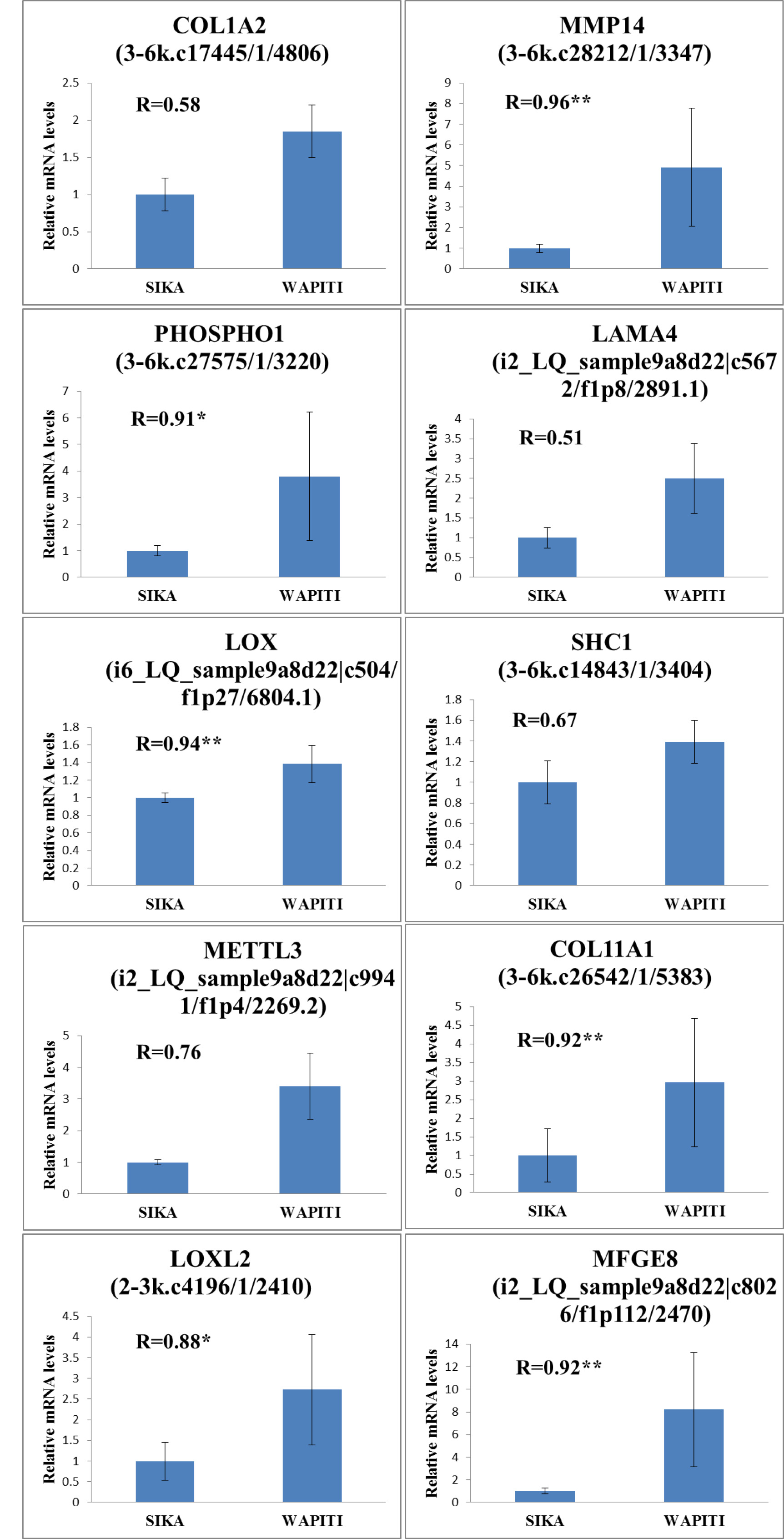

Supplement: Supplementary file 1 [file animals-12-02203-s001.zip › animals-1855021-supplementary/Figure S2.jpg]

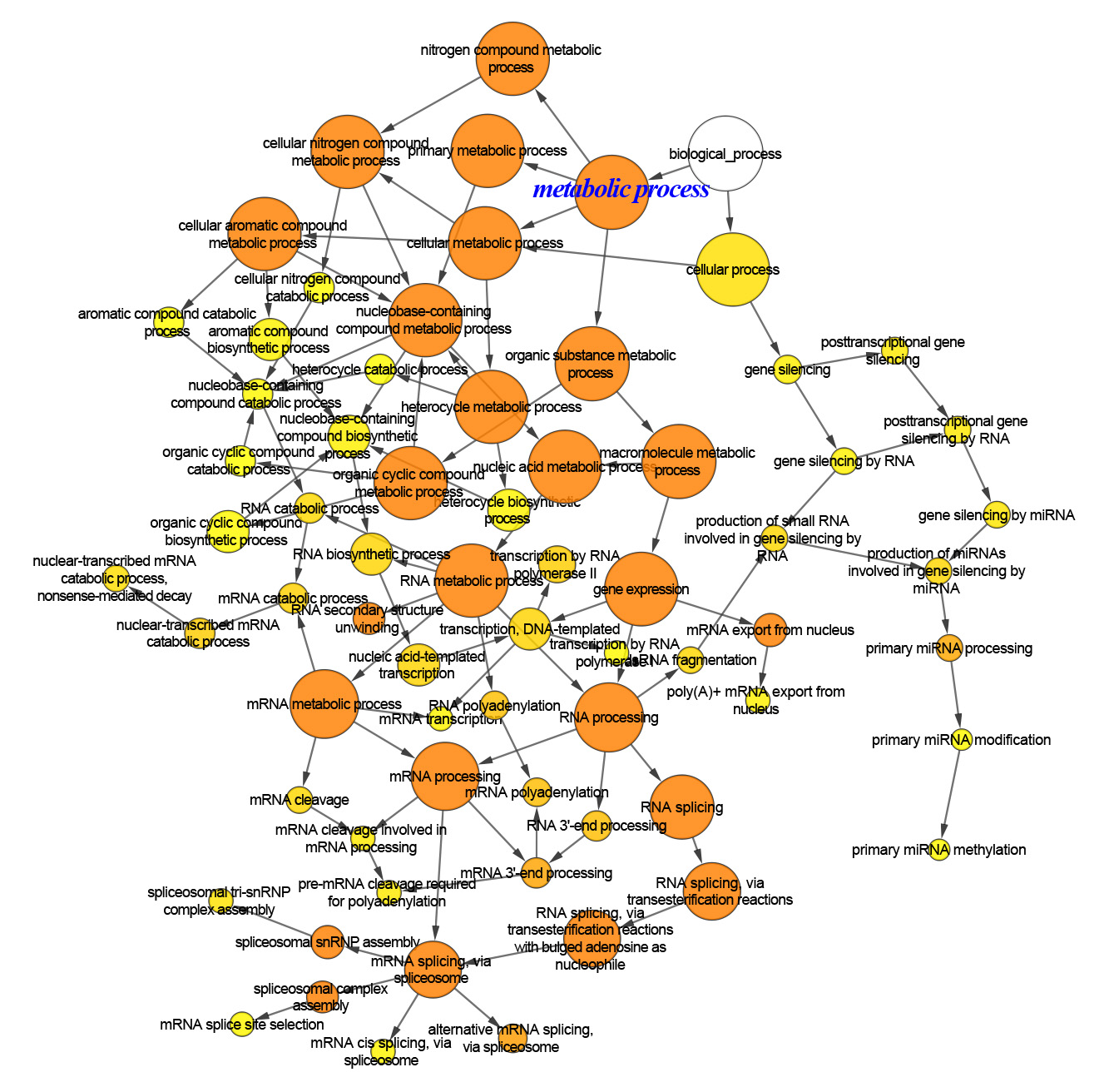

Supplement: Supplementary file 1 [file animals-12-02203-s001.zip › animals-1855021-supplementary/Figure S3.jpg]
